# Development of multiplex reverse transcription-polymerase chain reaction for differentiation of strains of infectious bursal disease virus and primary screening of the virus in Thailand

**DOI:** 10.14202/vetworld.2021.3105-3110

**Published:** 2021-12-14

**Authors:** Nataya Charoenvisal

**Affiliations:** Avian Health research Unit, Department of Veterinary Medicine, Faculty of Veterinary Science, Chulalongkorn University, Bangkok 10330, Thailand

**Keywords:** infectious bursal disease virus, multiplex reverse transcription-polymerase chain reaction, restriction fragment length polymorphism, strain, Thailand

## Abstract

**Background and Aim:**

A new set of primers (400 base pairs partial of VP2) was designed and used for the infectious bursal disease virus (IBDV) screening test. Using this new primer set, the enzymes *MboI* and *BstNI* were unable to differentiate the field and vaccine strains. As a result, a new simple, cheap, and appropriate tool for strain differentiation is required. The objective of this study was to develop the appropriate restriction fragment length polymorphism (RFLP) and multiplex reverse transcription-polymerase chain reaction (RT-PCR) for the differentiation of classic IBDV (cIBDV) strains and very virulent IBDV (vvIBDV) strains in Thailand.

**Materials and Methods:**

Ninety seven bursa of Fabricius from 16 farms were collected from farms in the eastern and central regions of Thailand. RT-PCR screening showed that 82 samples were positive for IBDV and 15 samples were negative. Then, selected samples were sequenced from each farm with a positive test.

**Results:**

The sequencing results showed that samples from six of the farms were vvIBDV and samples from the other six farms were cIBDV. Although the whole genome sequencing was incomplete, both the sequencing results of segment A and segment B showed high similarity between cIBDV and vvIBDV. Restriction enzyme cutting site and primers for multiplex RT-PCR were hard to design. An RT-PCR-RFLP method was developed, but it failed to differentiate IBDV strains. However, the multiplex RT-PCR was able to differentiate cIBDV from vvIBDV. Four primers were used in the multiplex RT-PCR.

**Conclusion:**

These four primers were used together in one reaction at an annealing temperature of 45°C. Therefore, multiplex RT-PCR is a less complicated, cheaper, and less time-consuming method for the differentiation of cIBDV and vvIBDV strains.

## Introduction

Infectious bursal disease (IBD) or Gumboro disease is a viral infectious disease that results in economic loss to the poultry industry worldwide. The disease is caused by IBD virus (IBDV). In chicken, the virus has a high mortality rate, can decrease production, and cause immunosuppressive effects [1]. IBDV has been of concern in the industry since the 1960s and still affects poultry health in many countries, including Thailand.

The IBDV genome consists of two segments. Segment A, size 3.2 kb, has two overlapping open reading frames (ORFs). The first ORF encodes one non-structural protein, VP5. The second ORF encodes three major structural proteins: VP2, VP3, and VP4. Segment B, size 2.9 kb, encodes the VP1 protein [2]. As VP2 is the most variable gene, it was chosen to identify IBDV strains [3-5]. IBDV is classified into seven genogroups by the hypervariable region of the VP2 protein. Genogroup 1 is for predominantly classic strains (classic IBD virus [cIBDV]). Genogroup 2 is for predominantly variant strains (vaIBDV). Genogroup 3 is predominantly very virulent IBDV (vvIBDV) strains. Genogroup 4 is for the viruses mostly found in Latin America, Japan, and the United Arab Emirates (UAE). Genogroup 5 is for the viruses found in Mexico and Southeast United States. Genogroup 6 is for viruses found in Saudi Arabia, Italy, and Russia. Genogroup 7 is the V877, found in Australia [6]. In Thailand, cIBDV, which is classified as genogroup 1 and vvIBDV, which is classified as genogroup 3, have been reported [7].

Sequencing and quantitative real-time reverse transcriptase-polymerase chain reaction (qRT-PCR) methods are accurate molecular tools for the identification of IBDV strains [4,5]. However, sequencing is time-consuming and qRT-PCR is expensive. In 1998, the RT-PCR analysis of 734 base pairs of the partial VP2 gene followed by restriction fragment length polymorphism (RFLP) analysis or RT-PCR-RFLP was developed to differentiate the field IBDV from vaccine strain. The enzymes *Mbo*I and *Bst*NI were used to create the RFLP [8]. This method was simple and has been applied in some laboratories in Thailand. Nevertheless, the primer set of 734 base pairs of partial VP2 was not specific to recent Thai IBDV isolates. Consequently, a new set of primers (400 base pairs partial of VP2) was designed and used for the IBDV screening test. Using this new primer set, the enzymes *Mbo*I and *Bst*NI were unable to differentiate the field and vaccine strains. As a result, a new simple, cheap, and appropriate tool for strain differentiation is required.

The objective of this study was to develop an appropriate RFLP and multiplex RT-PCR for the differentiation of cIBDV and vvIBDV in Thailand.

## Materials and Methods

### Ethical approval

Bursa of Fabricius was collected during routine services at the farms. However, the biosafety of virus used in this study was approved by Faculty of Veterinary Science Biosafety Committee Chulalongkorn University (protocol number IBC1931001).

### Study period and location

The study was conducted from April 2017 to March 2021 and was conducted in eastern and central parts of Thailand.

### Sample collection

From 16 farms in the eastern and central parts of Thailand, 97 bursa of Fabricius were collected from chickens with suspected IBDV infection. The fresh tissues were kept in an icebox for 1-2 days during shipping to the laboratory. Subsequently, the fresh tissues were stored at −80°C until the investigation.

### RT-PCR for IBDV screening

All samples were screened for IBDV using RT-PCR analysis. First, each bursa of Fabricius, representing one sample, was homogenized. Then, viral RNA was extracted following the commercial test kit protocol (Geneaid Total RNA mini kit [Tissue], Taiwan). All RNA samples were screened by RT-PCR using primers specific for 400 bp of the partial VP2 gene. The forward primer was IBDVP2_F658 (5′-TACCAATTCTCATCACAGTACCAA-3′) and the reverse primer was IBDVP2_R1039 (5′-CGGAGGGCCCCTGGATAGTT-3′). The positive samples were retained throughout the study, whereas the negative samples were discarded or used as a negative control. From all farms tested, 15 positive samples were selected, with at least one sample from each IBDV-positive farm (Table-1). The samples were then sequenced for 400 base pairs (a partial part of the VP2 gene) and were classified.

**Table 1:** Bursa of Fabricius samples collected from farms in Thailand from April 2017 to October 2019.

Farm	No. of total samples (No. of sequence sample)	Bursal lesion	IBDV screening test (Strain classification result)
A	10 (2)	Redness, swelling, inflammation, petechial hemorrhage, and debris found inside the bursa	All samples were positive (vvIBDV)
B	5 (1)	Redness, swelling, inflammation, petechial hemorrhage, and debris found inside the bursa	All samples were positive (vvIBDV)
C	1	Swelling	Negative
D	11	No lesion	All samples were negative
E	2	Bursa was smaller than usual	All samples were negative
F	5 (1)	Redness, swelling, inflammation, petechial hemorrhage, and debris found inside the bursa	All samples were positive (cIBDV)
G	7 (1)	Redness, swelling, inflammation, petechial hemorrhage, and debris found inside the bursa	All samples were positive (cIBDV)
H	7 (1)	Redness and swelling	All samples were positive (vvIBDV)
I	5 (1)	Redness, swelling, inflammation, petechial hemorrhage, and debris found inside the bursa	All samples were positive (vvIBDV)
J	17 (2)	Redness, swelling, inflammation, petechial hemorrhage, and debris found inside the bursa	All samples were positive (cIBDV)
K	1	Redness	Negative
L	10 (1)	Redness, swelling, inflammation, petechial hemorrhage, and debris found inside the bursa	All samples were positive (vvIBDV)
M	2 (1)	Samples were already homogenate at the farm	All samples were positive (cIBDV)
N	2 (1)	Redness	All samples were positive (cIBDV)
O	10 (2)	Redness	All samples were positive (vvIBDV)
P	2 (1)	Redness, swelling, and petechial hemorrhage	All samples were positive (cIBDV)

IBDV=Infectious bursal disease virus, vvIBDV=Very virulent infectious bursal disease virus, cIBDV=Classic infectious bursal disease virus

### Development of RFLP for differentiating IBDV strains

Two vvIBDV and two cIBDV Thai isolates were selected. The isolates were sequenced for 400 bp (a part of the VP2 gene) and classified into each strain in the screening part. The whole-genome sequence of these isolates was analyzed. The whole genome of IBDV is composed of segment A and segment B. Segment A has 3260 base pairs and segment B has 2816 base pairs. The different points in each strain were compared and an appropriate region for RT-PCR-RFLP was designed. Then, the designed RT-PCR-RFLP was tested with these four selected isolates and IBDV vaccine for optimal time, temperature, and ratio of substance until the result was stable and able to identify the right strains.

In brief, PCR products from forward primer IBDVP2_F658 and reverse primer IBDVP2_R1039 (primers for screening test) were used as samples. The PCR products (11 μL) were mixed with NEBuffer™ (BioLab Inc., USA) 2.5 μL, nuclease-free water 11 μL, and CviQi enzyme (BioLab Inc.) 0.5 μL, and then incubated at 25°C for 15 min.

### Development of multiplex RT-PCR to differentiate IBDV strains

The set of samples used for the RFLP development part was used again here. The sequences of the selected strains were compared and used to design an appropriate set of primers to differentiate between strains. The primers had to be able to operate in the same PCR condition without matching to any other sequences. The PCR products of each strain had to result in different numbers of DNA base pairs. Then, the designed primers were tested with the four selected isolates and vaccines for the optimal time, temperature, and substance ratio until the results were stable and identification of the strains was achieved.

### Sequencing of IBDV

One or two samples from each farm with a positive result were selected and used for sequence analysis (400 bp of the partial VP2 gene). The sequence analysis was performed using Sanger sequencing technique; the primers are shown in Table-2. The sequence results were compared with the reference strains in GenBank using the Maximum Composite Likelihood model [9] conducted by MEGA X [10]. The reference sequences in GenBank included the following: Strain or isolate STC (D00499), Lukert (AY918948), D78 (AF499929), variant E (AF133904), dIBDV (KT336459), Henan (KT884486), HK46 (AF092943), OKYM (D49706), HLJ-0504 (GQ451330), and UPM766/2018 (MT505341).

**Table 2:** Primer designed for whole-genome sequencing.

Primer name	Primer sequence (5’→3’)	Size	Position
IBDV-A-FB1	GGATACGATCGGTCTGACCCC	21	1-21
IBDV-A-R246	GAACTTGTAGTTCCCATTGCTCTG	24	223-246
IBDV-A-F116	GGTCAGAGACCTCGACCTACAAT	23	116-138
IBDV-A_R728	CCAACGCTGAGACTTGTGATGG	22	707-728
IBDVP2_F658	TACCAATTCTCATCACAGTACCAA	24	658-682
IBDVP2_R1039	CGGAGGGCCCCTGGATAGTT	20	1039-1049
IBDV-A_F976	TCATGGTCAGCAAGTGGGAGC	21	976-996
IBDV-A-R1499	GGGAACAATGTGGAGACCACCG	22	1500-1521
IBDV-A-F1431	CTCCCCTGAAGATTGCAGGAGC	22	1431-1452
IBDV-A_R1846	CCCCATCTGGAGCATATCCATA	22	1825-1846
IBDV-A_F1792	TCCTTCATACGAACTCTCTCCG	22	1792-1813
IBDV-A_R2590	CCTCCATCTTCTTTGAGATCCG	22	2569-2590
IBDV-A_F2504	AGCAGGCTACGGAGTGGAGG	20	2505-2524
IBDV-A_R3137	TACCAAGGGGACCCGCGAAC	20	3117-3136
IBDV-B_F01	GGATACGATGGGTCTGACCCT	21	3-23
IBDV-B-R520	CTTCCGGAAGCTGGAGAAATAG	22	520-541
IBDV-B-F410	CCATTGGTGACCAAGAGTACTTCC	24	410-433
IBDV-B_R921	TTTCTCCTTTGGTGCGACCAAC	22	898-921
IBDV-B_F875	CAAGTCATCAAGTGGACTGCCC	22	875-896
IBDV-B-R1387	CCGCCTGCATGTGTTGACG	19	1387-1405
IBDV-B-F1357	GACCTAGAGAAGGGGGAGGC	20	1357-1376
IBDV-B-R2344	CTTTGGCCTTTACTGCATCTTG	22	2344-2365
IBDV-B-F2286	CAGGAATGAAGCCGGACTGAGTGG	24	2286-2309

IBDV=Infectious bursal disease virus

## Results

### Sample collection and RT-PCR for IBDV screening

Ninety-seven bursal of Fabricius samples were collected from 16 farms in central and eastern parts of Thailand (Table-1). All samples were homogenized, processed to extract RNA, and then screened by 400 bp RT-PCR. The RT-PCR results showed that 82 samples from 12 farms were positive and 15 samples from four farms were negative.

### Sequencing of IBDV

Of the 12 farms with a positive test, samples from six farms were cIBDV positive and samples from the other six farms were vvIBDV positive. The results showed that from March 2017 to October 2019, both cIBDV and vvIBDV were circulating in Thailand (Table-1). Primers were designed for whole genome sequencing and the sequences of four samples were examined. Samples 1 and 2 were representative samples of cIBDV and were selected from farm F and G, respectively. Samples 3 and 4 were representative samples of vvIBDV and were selected from farm H and I, respectively.

However, the whole genome sequences were not completed. In segment A, only nucleotide sequences of sample 3 were completed (3260 base pairs); nucleotides 1-245 were incomplete in samples 1 and 2, and nucleotides 1-249 of sample 4 were incomplete. In segment B, 30-500 base pairs of all four samples were randomly incomplete, although the 2816 base pairs were sequenced.

Segment A from samples 1-4 was compared to the reference sequences in GenBank, starting from nucleotide base 250 to 3,169. The result showed that samples 1 and 2 were 97.01%-98.79%, similar to isolate STC, Lukert, and D78, which were representative of cIBDV. These samples were 94.44%-96.90% similar to vaIBDV (variant E), dIBDV (genotype 4), and vvIBDV (Henan, HK46, OKYM, HLJ-0504, and UPM766/2018). Samples 3 and 4 were 96.98%-97.70% similar to the reference vvIBDV (Henan, HK46, and OKYM) and 98.51%-99.24% similar to isolate HLJ-0504 and UPM766/2018 (vvIBDV isolates from China and Malaysia, respectively). Samples 3 and 4 were 94.26%-95.15% similar to the cIBDV, 94.97%-94.51% similar to vaIBDV, and 93.05%-93.09% similar to dIBDV (genotype 4) (Table-3).

**Table 3:** Comparing percent similarity of segment A (nucleotide 250-3169) between samples 1 and 4 and reference sequences from the GenBank.

Strains	Sample 1	Sample 2	STC	Lukert	D78	Variant E	dIBDV	Henan	HK46	OKYM	HLJ-0504	UPM766	Sample 3
Sample 1													
Sample 2	99.83											
STC	98.79	98.61											
Lukert	97.12	97.01	96.79										
D78	97.87	97.77	97.66	96.75									
Variant E	96.90	96.80	96.54	95.94	96.69								
dIBDV	95.70	95.29	95.43	94.72	95.14	94.34							
Henan	95.41	95.30	94.95	94.08	95.07	94.51	93.05						
HK46	95.49	95.38	95.11	94.13	95.26	94.59	93.18	97.84					
OKYM	95.79	95.68	95.48	94.44	95.41	94.97	93.45	98.05	99.03				
HLJ-0504	95.06	94.96	94.88	93.97	94.77	94.77	92.88	96.80	97.27	97.63			
UPM766	94.47	94.44	94.35	93.39	94.12	93.59	92.23	96.21	96.48	96.84	98.40		
Sample 3	95.26	95.15	95.06	94.27	95.15	94.47	93.09	97.01	97.41	97.70	99.24	98.54	
Sample 4	95.23	95.11	95.06	91.30	95.14	94.51	93.05	96.98	97.38	97.67	99.20	98.51	99.97

### RFLP attempt for differentiating IBDV strains

Segment A was chosen for the strain differentiation test because it was closer to a full sequence. Moreover, the most highly variable region (the VP2 gene) was located in segment A. The restriction enzyme was designed from the 400 base pairs of the VP2 gene (from 658 to 1039) as this part of all four isolates was detectable. The enzyme CviQi, which cuts the nucleotide sequence at 5′---G/TAC---3′ and 3′---CAT/G---5′, was selected as a restriction enzyme. The cIBDV strain was expected to show a band of 300 base pairs, and the vvIBDV strain was expected to show a band of 400 base pairs.

Therefore, the enzyme was not appropriate as it resulted in a band of 400 base pairs in all samples. Other enzymes restriction sites were used to compare within the 400 base pair sequence, but there was no enzyme that could cut the different parts of the two different strains.

### Development of multiplex RT-PCR for differentiating IBDV strains

Segment A was more variable than segment B, so the primers to differentiate IBDV strains were designed from segment A (Table-4). From this set of primers, the cIBDV samples were expected to show a specific band of 700 base pairs and the vvIBDV samples were expected to show a specific band of 387 base pairs. Other viruses, such as Newcastle disease virus (NDV) and infectious bronchitis virus (IBV), were not expected to show any specific bands in this reaction.

**Table 4:** Primers designed for multiplex RT-PCR.

Primer name	Primer sequence (5’→3’)	Size	Position
MPC-F1	TTACAACTACTGCAGGCTAGTG	22	415-436
MPC-R1	CGACCACGACATCTGATCC	19	1096-1114
MPVV-F1	TTAAACGGAACCATAAACG	19	488-511
MPVVR1	CACAAGTTCTCCCCCGAT	18	854-875

RT-PCR=Reverse transcription polymerase chain reaction

**Figure-1:**
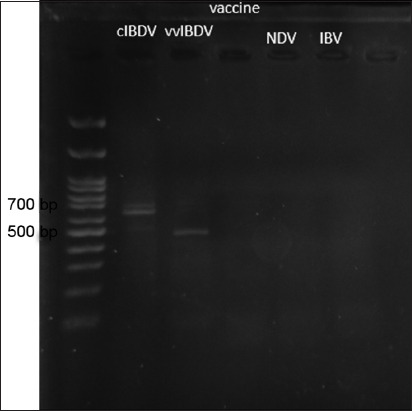
Result of multiplex reverse transcription-polymerase chain reaction showed a specific band of classic infectious bursal disease virus (BDV) at 700 base pairs and a specific band of very virulent infectious BDV at 500 base pairs. There was no specific band in other viruses.

This primer set could be mixed in a single PCR mixture without any binding to other primers in the set; moreover, the primers worked at the same annealing temperature (45°C). The results showed specific bands for cIBDV at 700 base pairs and for vvIBDV at 500 base pairs, as expected (Figure-1). The IBDV vaccine did not give a positive result when using a set of primers. NDV and IBV could not be detected in this test. Therefore, the materials used in this test can be considered to be specific and sensitive.

## Discussion

IBD is a highly contagious disease in young chickens and causes economic loss in the poultry industry. New technologies are continuing to improve vaccine development and diagnostic techniques. However, the new technologies usually require expensive equipment and materials. If problems are noticed on the farm, the farmer has to send the samples to a laboratory to differentiate the IBDV strain and consequently plan to adapt the vaccination program. Fast diagnostic results and strain identification will benefit the prevention and control program [11]. Nowadays, larger farms have developed diagnostic tools to serve their own farms and customer (the contact farms). Hence, less expensive and less complicated diagnostic methods are still advantageous.

The result of this study showed that at least two genogroups of IBDV (genogroup 1 [cIBDV] and genogroup 3 [vvIBDV]) were found circulating in Thailand. The whole-genome sequencing of four samples was performed to study the appropriate site to differentiate IBDV strains. Unfortunately, only partial sequences of both segments A and B were successfully determined. Based on the homology result of segment A, samples 1 and 2 were classified as genogroup 1 because they were most similar to STC strain isolated in the United States [12]. Samples 3 and 4 were classified as genogroup 3 as they were highly similar to the isolates from this genogroup. Moreover, they were highly similar to isolate UPM766/2018, which was reported in Malaysia [13] and HLJ-0504, which was reported in China [14]. There were no other variant IBDV strains found in this study. Interestingly, vaIBDV was recently reported in Malaysia and China [13,15]. Therefore, considering geographic territory, the variant strain may already have been circulating in Thailand. This result indicates that surveillance of IBDV must be performed continuously. Unfortunately, Jackwood and Sommer-Wagner (2007) are the only authors to report the nucleotide sequences of IBDV in Thailand.

The next part of the study was to develop the RFLP technique or RT-PCR-RFLP and multiplex RT-PCR. Although the whole genome sequence was incomplete, the nucleotide sequence of cIBDV and vvIBDV was highly similar in both segment A and segment B (95.21%). The point mutations were found throughout each segment, but only 1-2 base pairs of each point were different. Therefore, the restriction enzyme and primer for multiplex RT-PCR were difficult to develop. In our study, the RT-PCR-RFLP failed to differentiate cIBDV and vvIBDV. The result showed the same band at 400 base pairs, which meant that the selected restriction enzyme could not cut both cIBDV and vvIBDV samples. Therefore, if the whole genome sequence is completed, a longer part of the sequence will be selected and a more appropriate enzyme can be chosen; this may allow the RT-PCR-RFLP to differentiate the IBDV strains.

In this study, the RT-PCR-RFLP method could not be developed. However, the multiplex RT-PCR showed good results. It was able to differentiate cIBDV from vvIBDV, showing specific bands at 700 base pairs and 500 base pairs, respectively. The primers were able to be placed in the same reaction and used the same annealing temperature. In addition, the multiplex RT-PCR was less time-consuming, less complicated, and cheaper than the RT-PCR-RFLP technique. Therefore, multiplex RT-PCR may be suitable for farmers to use on the farm.

## Conclusion

Although multiplex RT-PCR is not a new method, it is easy to apply in small laboratories and does not require high skill or expensive equipment. However, further study of the IBDV strains circulating in Thailand should be performed with more number of samples to benefit the prevention and control of IBDV.

## Authors’ Contributions

NC: Collected the bursa of Fabricius from farms A, C, D, E, H, K, M, N, O, and P, designed experiment, processed all the laboratory work, concluded and wrote the manuscript and approved the final manuscript.
